# The functional role of long non-coding RNAs and epigenetics

**DOI:** 10.1186/1480-9222-16-11

**Published:** 2014-09-15

**Authors:** Jinneng Cao

**Affiliations:** 1 Department of respiratory medicine, Fuyong People’s Hospital, Baoan District, Shenzhen 518103, Guangdong, People’s Republic of China

**Keywords:** lncRNAs, Epigenetics, Transcriptional repression, Chromatin

## Abstract

Long non-coding RNAs (lncRNAs) are non-protein coding transcripts longer than 200 nucleotides. The post-transcriptional regulation is influenced by these lncRNAs by interfering with the microRNA pathways, involving in diverse cellular processes. The regulation of gene expression by lncRNAs at the epigenetic level, transcriptional and post-transcriptional level have been well known and widely studied. Recent recognition that lncRNAs make effects in many biological and pathological processes such as stem cell pluripotency, neurogenesis, oncogenesis and etc. This review will focus on the functional roles of lncRNAs in epigenetics and related research progress will be summarized.

## Introduction

Messenger RNA (mRNA) is the RNA that carries information from DNA to the ribosome for protein synthesis. And yet, many RNAs do not code for proteins in eukaryotes. There are RNAs that lack an apparent open reading frame (ORF) of 300 nt or longer. They do not encode a protein product thus classified as putative noncoding RNAs [1-3]. Long non-coding RNAs (lncRNAs) are molecules longer than 2 kb in length with a coding potential of less than 100 amino acids, or non-protein coding transcripts with the length of longer than 200 nucleotides (nt) [1,4-6]. For this definition, it somewhat arbitrary could not distinguish lncRNAs from small regulatory RNAs. Now, there have been identified far greater amounts of lncRNAs than protein coding genes [7-9]. A majority of annotated eukaryotic protein-coding ORFs were characterized with high level of phylogenetic diversity and the conservation. And the level of its conservation and the rate of synonymous to no synonymous substitutions were applied as additional criteria. It also applied for distinguishing the protein-coding transcripts containing bona fide functional ORFs from non-coding transcripts among novel RNAs [1-3]. As we know, there is an underlying dogma of molecular biology that the purpose of RNA is to direct the assembly of proteins from amino acids. However, a few exceptions to this paradigm were explored (such as ribosomal RNA and transfer RNA), which were functional RNA macromolecules that did not code for protein [10].

It has been reported that about 20% of transcription progress across the human genome would be associated with protein-coding genes [11]. The fact indicates that lncRNAs is at least four-times longer than coding RNA sequences [5]. However, it may be large-scale complementary DNA (cDNA) sequencing projects to reveal the complexity of transcription, such as FANTOM (Functional Annotation of Mammalian cDNA) [12].

RNA is an information encoding molecule with high flexibility and high-fidelity. It has also been characterized with easy activation, modification, transportation, and degrasion. Thus, RNA is considered as an integrative character of both the digital lexicon of DNA and the analog language of proteins. It is also a dynamic participant of DNA and protein molecules in performing cellular activities.

**Figure 1:**
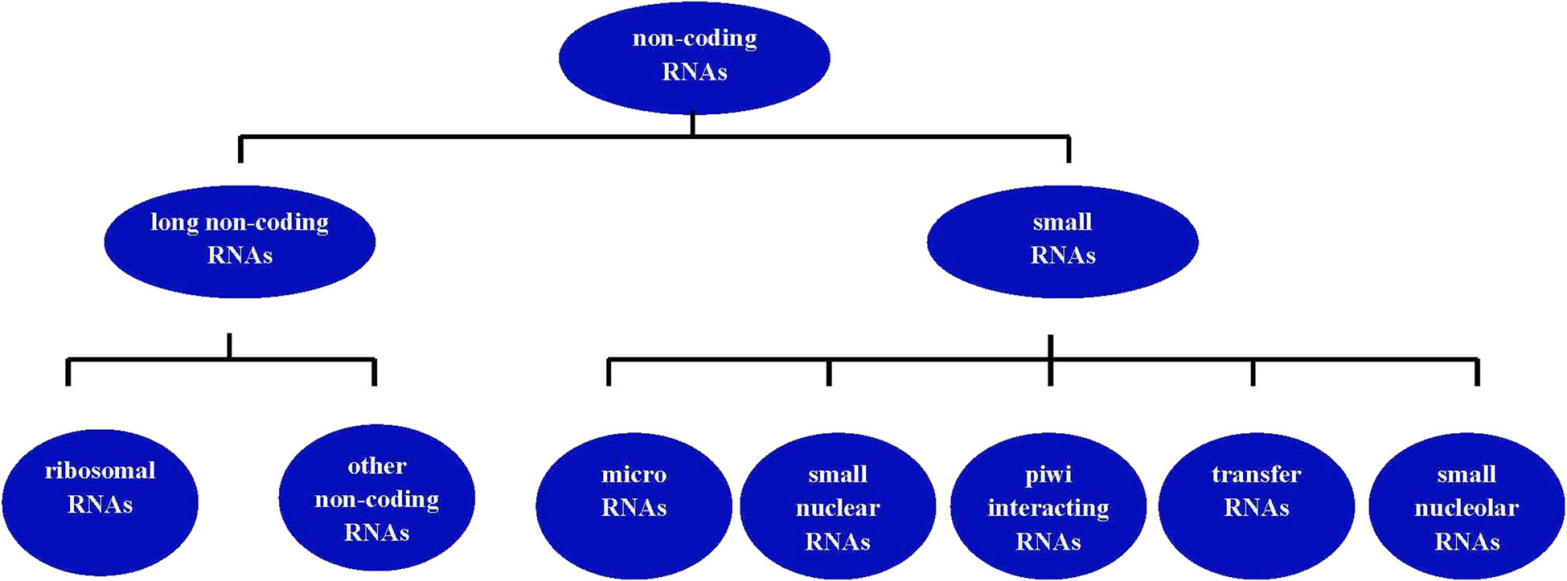
Classification of lncRNAs based on the sizes.

According to taxonomic, non-coding RNAs (ncRNAs) are composite of the familial "housekeeping" RNAs and regulatory RNAs in recent intensive studies. There are many different sizes of NcRNAs and for this reason they have been divided into small and long classes: small ncRNAs (sncRNA) being less than 200 nt and lncRNA greater than 200 nt to over 100 kb in length [13]. The current cut-off has been arbitrary and corresponds to specific biochemical protocols. Most categories of small infrastructural or regulatory RNAs have been excluded (tRNAs, snRNAs, miRNAs, siRNAs, piRNAs, tiRNAs, spliRNAs, sdRNAs and others) (Figure 1) [10].

It is predicted that there are thousands of lncRNAs in the mammalian transcriptome [2,14-17]. No strict minimal size is required for classifying a noncoding transcript as a "long" non-coding RNA and there were many lncRNAs with thousands of nucleotides [18]. There are no clear-cut, uniformly available criteria for determining a non-coding character of an RNA [2,19,20]. The widely accepted method for distinguishing protein-coding and non-coding RNAs among novel transcripts has analyzed the ORFs in each transcript as a primary criterion [21-24].

Epigenetics associated with a gene activity state that may be stable over long periods of time, persist through many cell divisions, or even be inherited through several generations and all without any variations to the primary DNA sequence [25-27]. What is the relationship of lncRNAs and epigenetics? In this review, the recent studies will be included and we have tried to demonstrate the function of lncRNAs and its influences on epigenetics.

### The function of lncRNAs

**Figure 2:**
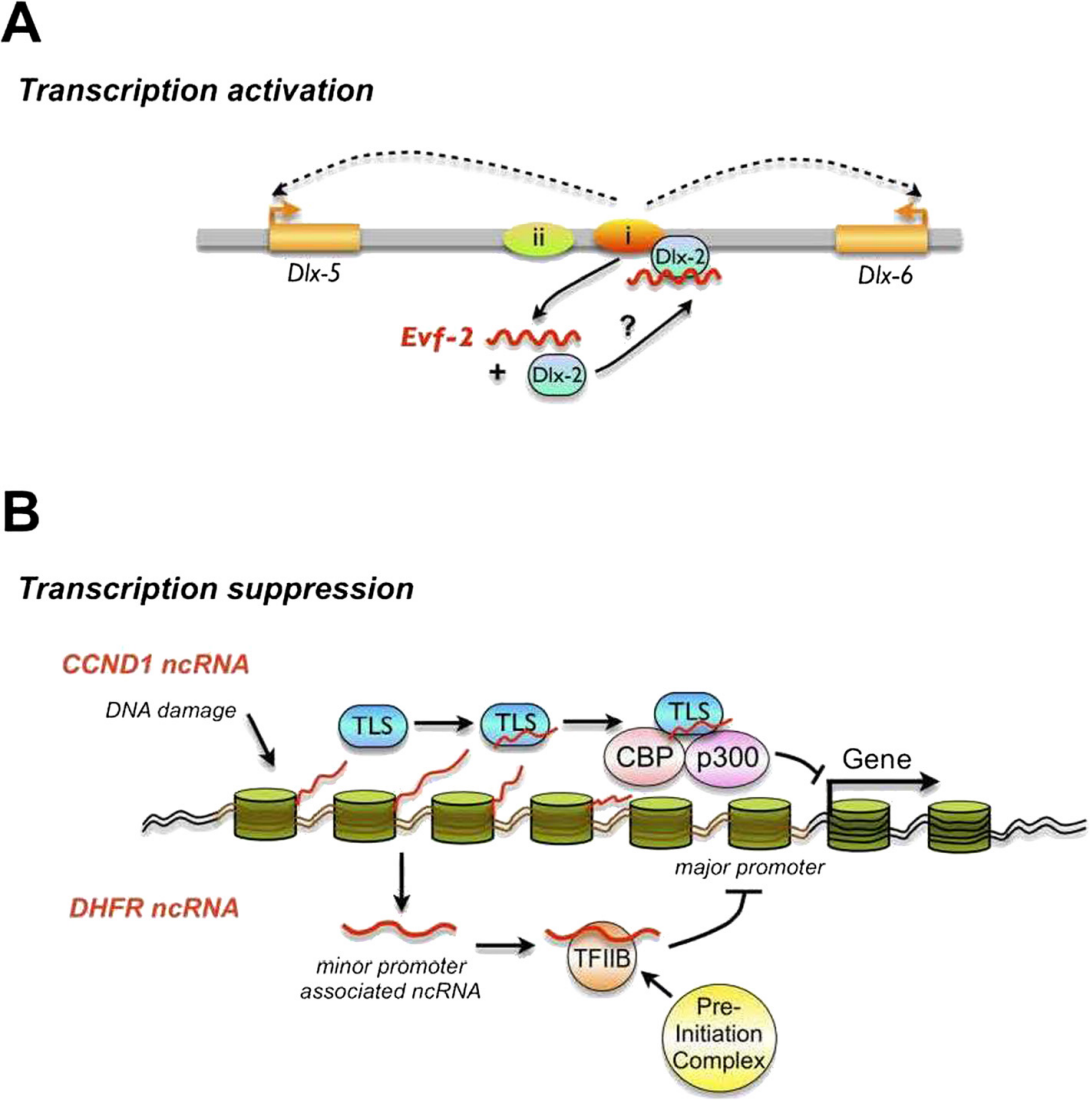
**Long non-coding RNA-mediated transcription regulation. A**. Transcription activation by lncRNA. In this example, *Evf*-*2* is transcribed from an ultra-conserved enhancer and forms a stable complex with Dlx-2, which in turn activates Dlx-2 as a transcriptional enhancer. **B**. Transcription suppression by lncRNA. Top: in response to DNA damage, lncRNAs are transcribed from the 5’-upstream region of the CCND1 gene and recruit the RNA-binding protein TLS to modulate CBP and p300 to inhibit CCND1 transcription. Bottom: LncRNA transcribed from the upstream of the minor promoter of DHFR gene competes with transcription factors to inhibit the major promoter transcription in quiescent cells.

LncRNAs were considered as non-functional junk initially. And now, their presence and significance have still being debated [28-30]. It is now apparently observed that many lncRNAs are the key regulators of transcriptional and translational output and therefore make effects on cell identify and function (Figure 2) [17,31-33]. However, different from general mRNAs exported to the cytoplasm for translation, many lncRNAs are now known to be restrained in various sub-nuclear compartments [3,34,35], which suggesting that such RNAs may have a potential function in the compartment where they are located.

**Figure 3:**
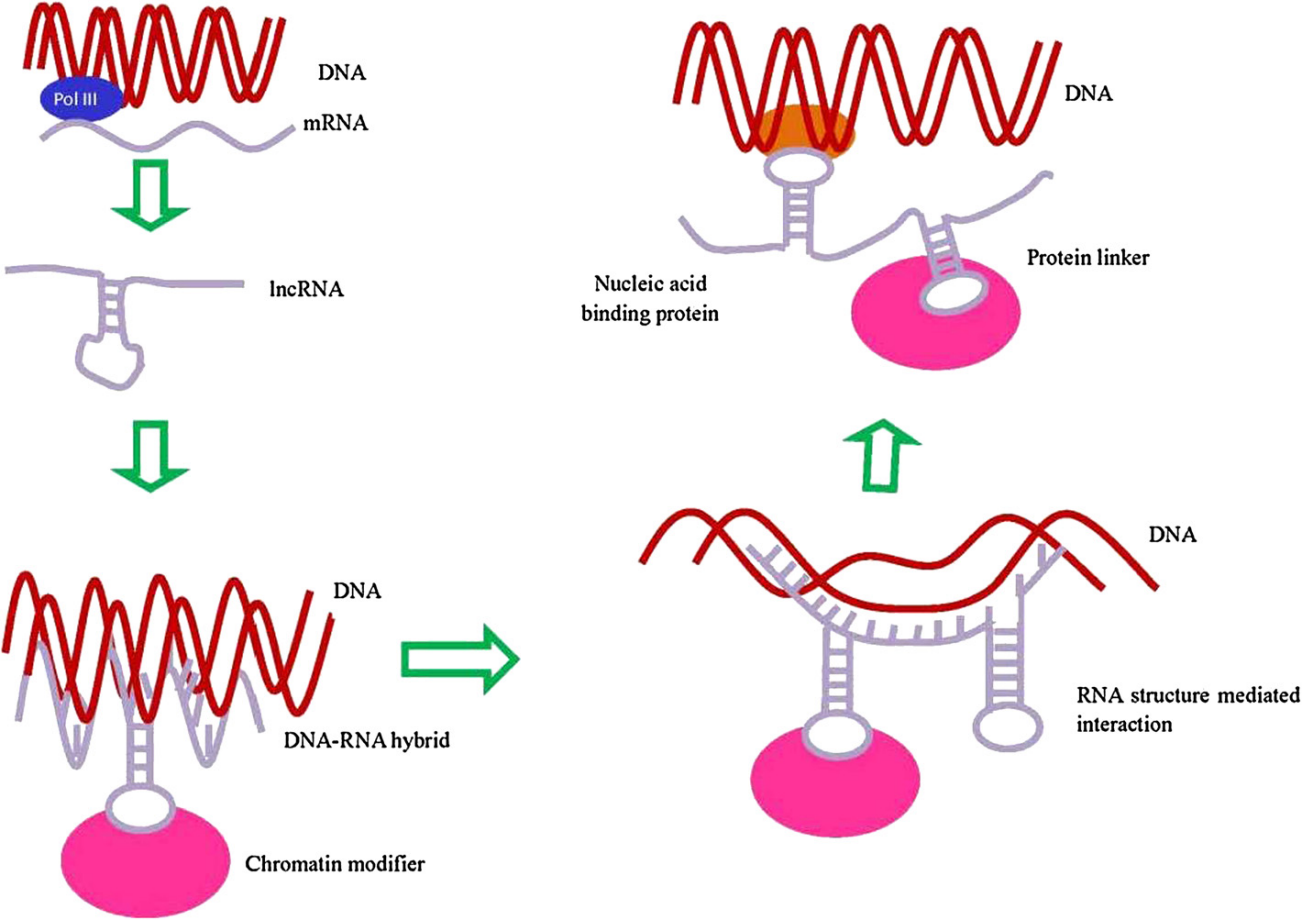
**Possible lncRNA targeting mechanisms.** Based on these potential mechanism, lncRNAs may play critical regulatory roles in diverse cellular processes such as chromatin remodeling, transcription, post-transcriptional processing and intracellular trafficking [16,19,31,36-40].

Studies have shown that lncRNAs play critical regulatory roles in diverse cellular processes such as chromatin remodeling, transcription, post-transcriptional processing and intracellular trafficking [16,19,31,36-40] (Figure 3). LncRNAs could be a highly abundant, rapidly evolving class of cellular factors with a wide range of cellular functions [2,19,36,41].

### Imprinting

Imprinting has been a significant process in identifying special nucleic acid and protein, such as DNA methylation and histone modification. LncRNAs have been found to participate in imprinting processes. It means lncRNAs influence the monoallelic expression of a gene according to its parents of origin. More than 200 lncRNAs were found to participate in imprinting processes. Depending on their parental origins, differentially methylated regions unmethylated DNA imprinting control regions (ICRs), resulting in specific expression of nearby lncRNAs and suppressing neighboring genes in *cis*[42].

The H19 lncRNA-MBD1 complex could interact with histone lysine methyltransferases. Therefore, it could work by bringing repressive histone marks on the differentially methylated regions of the three direct targets of the H19 gene [43].

Airn and Kcnq1ot1/LIT1 (Kcnq1 opposite transcript 1, or long QT intronic transcript 1) are examples of lncRNAs that cause suppression of paternally inherited genes. In particular, Kcnq1ot1/LIT1 is involved in the repression of several protein-coding genes in *cis* by interacting with repressive chromatin modifying complexes [44,45]. Kcnq1ot1/LIT1 is an imprinted region contains at least eight genes that are expressed exclusively or preferentially from the maternal allele [46]. Kcnq1ot1/LIT1 acts as an organizer on a tissue/lineage-specific nuclear domain, involving in epigenetic silencing of the Kcnq1 imprinting control region [46-49].

### Developmental regulation

LncRNAs have played crucial roles in controlling gene expression during both developmental and differentiation processes. Furthermore, the number of lncRNA species will be higher in genomes of developmentally complex organisms. It highlights the significance of RNA-based levels of control in the evolution of multi-cellular organisms [50]. Expression of lncRNAs is dynamically regulated during male germline development. On the contrary, lncRNAs may function to regulate gene expression at both transcriptional and posttranscriptional levels based on both genetic and epigenetic mechanisms [51].

MEG3 (Gtl2) was a lncRNAs in human with the length of about 1.6 kb. There are a number of splice isoforms in MEG3 and it retains introns creating longer transcripts [52,53]. Recent studies have shown that Meg3 splicing isoform was silenced and in pituitary tumor, cancer cell growth would be inhibited by its ectopic expression. All these results suggested that Meg3 RNA acted as a growth suppressor [54]. Furthermore, MEG3 expression is not only associated with tumor grade, but also suppressing DNA synthesis and stimulating p53-mediated trans-activation in meningiomas cell lines [55]. Meg3 may play vital anti-tumor effects in tongue squamous cell carcinoma pathogenesis and represent potential prognostic biomarkers for stratification of patients with tongue squamous cell carcinoma [56].

### Diseases associated induction/derivation

More and more evidences have proved that lncRNAs play critical roles in various biological processes. The mutations and dys-regulations of these lncRNAs contribute to the development of many complex diseases, such as virus infection and carcinogenesis. Beta2.7 is the popular one being studied in virus infection. Generally, beta2.7 specifically binds and prevents the re-localization of essential complex I subunit GRIM-19 (gene associated with retinoid/interferon-induced mortality-19). In response to apoptotic stimuli, beta2.7 is responsible for stabilizing the mitochondrial membrane potential and mitochondrial ATP production. It also prevents metabolic dys-function, which will be essential for completing the virus’ life cycle [57,58]. The reactive oxygen species production will be reduced by the over-expression of beta2.7 RNA, thus the apoptosis could be inhibited [59,60]. LncRNAs have been strongly associated with cancer [61]. The expression of LncRNA PRINS (Psoriasis susceptibility-related RNA Gene Induced by Stress) will be in psoriatic epidermis and it will also be regulated by the proliferation and differentiation state of keratinocytes [62-64]. In keratinocytes, the expression of G1P3 is an anti-apoptotic protein with high expression in psoriasis and it will be regulated by lncRNA PRINS [65].

LncRNAPCAT-1, a target gene of polycomb repressive complex 2, has been implicated in disease progression by promoting cell proliferation [66]. The up-regulation of ANRIL (antisense non-coding RNA in the INK4 locus) is required for the expression of the tumor suppressors INK4a/p16 and INK4b/p15 in prostate cancer [67-69]. HOTAIR up-regulation is associated with poor prognosis in breast cancer, liver, colorectal, gastrointestinal and pancreatic cancers. Meanwhile, it also probably contributes to promote the tumor invasiveness and metastasis [70-75]. In human melanomas, approximately 50 genes have been partly regulated by hyper-methylation of CpG islands in their regulatory regions [76].

Compared with DNA or protein, RNA molecules are considered to be more efficiently couple bioenergetic requirements with information storage and processing [77]. Therefore, the advent of RNA based networks is thought to be responsible for fueling the explosive evolutionary innovations, which may characterize the human brain form and function [41,78,79]. The brain is a conspicuous consumer of energy resources. It is also a major consequence of cerebral ischemia for energy metabolism and exhaustion of adenosine triphosphate [80]. Brain development and function are tightly regulated by epigenetic mechanisms by gene expression modulation in response to intrinsic and extrinsic signals [81,82]. The lncRNAs are also dynamically expressed during pluripotency and differentiation in neural or glial cells [83,84]. The knock-down of four lncRNAs has been associated with neuronal differentiation. The function of these mentioned IncRNAs were mainly physically interacted with SOX2, PRC2 complex component, REST and SUZ12. The cellular differentiation fate will be altered from a neurogenic to a gliogenic program. The results suggested the functional role of the lncRNAs in neural cell fate specification [84-87].

### The versatile function of lncRNAs

**Figure 4:**
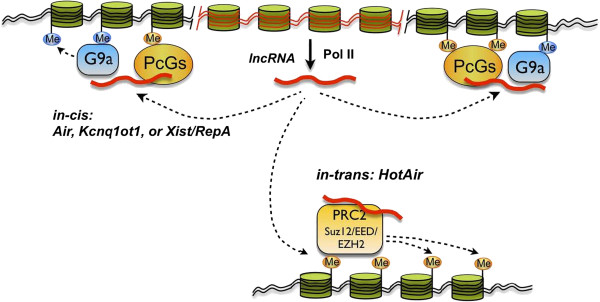
**Long non-coding RNA-mediated chromatin remodeling.** The lncRNAs, transcribed by RNA polymerase II could recruit transcriptional repressive complexes including PcGs and G9a to silence specific genomic regions, both in *cis* (top) and in *trans* (bottom) [31,96,97].

There are many different functions of lncRNA has been explored in latest few years. Besides of transcription regulation, there are also several versatile lncRNAs that have been evidenced, such as Kcnq1ot1, Airn, Xist and HOTAIR. Their function have mainly focused on regulating transcription of multiple target genes through epigenetic modifications [46,88-90]. For the insulin like growth factor 2 receptor (Igf2r) imprinted cluster, located on mouse chromosome 17, the expression of paternal-specific non-coding transcript antisense Igf2r RNA (Airn, 108 kb), is required for the silencing of three genes on the paternal allele. These genes have spread over a large genomic region spanning 400 kb [91]. On the mouse X-chromosome, expression of X-inactive specific transcript (Xist) of lncRNA from the designated inactive X-chromosome is essential for the silencing of inactive X-chromosome [87,92-94]. Some genes on the Homeobox D (HOXD) cluster are located over a 40 kb genomic region on human chromosome 2. These genes will be silenced by lncRNA HOTAIR, which is originated from the HOXC cluster on chromosome 12 [95]. On mouse chromosome 7, the potassium voltage-gated channel subfamily Q member 1 (Kcnq1) imprinted cluster spreads over a 1 Mb genomic region in embryos. Multiple genes are contained and it will be silenced on the paternal allele by the un-spliced lncRNA Kcnq1 overlapping transcript 1 (Kcnq1ot1, 91 kb) in *cis*. While, some lncRNAs, transcribed by RNA polymerase II, are able to recruit transcriptional repressive complexes including PcGs and G9a to silence specific genomic regions, both in *cis* (top) and in *trans* (bottom) [31,96,97] (Figure 4).

Spliced lncRNAs, compared with such un-spliced as single exon transcripts, intergenic and *cis*-antisense RNAs are more stable than those derived from introns [98]. The sub-cellular localization analysis indicates the location of lncRNAs is widespread in cell, with nuclear-localized lncRNAs more likely to be unstable [99].

### LncRNAs and epigenetics

Epigenetics is applied to describe the study of heritable variations in gene activity which is independent of DNA sequence variations in genetics. It is generally applied to refer to epigenetic modifications on the genetic material of one cell. Epigenetics is analogous to genomics and proteomics and it is the study focusing on genome and proteome of one cell [100]. Epigenetic modifications are reversible modifications on the DNA of one cell or histones that may affect gene expression without altering the DNA sequence [100].

### LncRNAs linked with epigenetics by DNA methylation

Chromatin is the combination of DNA and proteins that collectively make up the contents of cell nucleus [101,102]. Chromatin is in charge of DNA packaging, gene expression and DNA replication [103,104]. The mechanism of epigenomic control is generally considered at the level of chromatin [105-107]. Histones proteins can be chemically modified by as the process such as acetylation, methylation, sumoylation and ubiquitylation. The processes will result in structural variations in chromatin and the access of DNA will be allowed [108-112].

Recent findings reveal that lncRNAs are implicated in serial steps of cancer development [113]. These lncRNAs interact with DNA, RNA, protein molecules and/or their combinations. It acts as an essential regulator in chromatin organization, transcriptional and post-transcriptional regulation. Their mis-expression confers the cancer cell capacities for tumor initiation, growth, and metastasis. There is also a review demonstrating the roles of lncRNAs in cancer diagnosis and therapy. It reported expression profiles were different for numerous lncRNA in urothelial cancer [114]. The phenotype-specific expression and a potential mechanistic target were studied and it demonstrated that the IncRNAs may be prognostic biomarkers for this cancer. The LncRNAs such as up-regulation of HOTAIR could be associated with poor prognosis in breast cancer, liver, colorectal, gastrointestinal and pancreatic cancers. Meanwhile, it also probably contributes to promote the tumor invasiveness and metastasis [70-75].

There are CpG islands in the upstream region of the miR-375 gene and aberrant DNA methylation in this gene can be observed in specific melanoma stage [115,116]. Histone modification of DNA methylation is one vital epigenetic mechanism to regulate the expression of genes [117]. DNA methylation and histone modifications are epigenetic mechanisms leading to the deregulation of lncRNAs expression in cancers [118]. Epigenetic up-regulation of lncRNAs at 13q14.3 in leukemia is linked to the down regulation of *Cis*. It is a gene cluster that targets in NF-kB [119]. Normal melanocytes, keratinocytes and cell lines derived from stage one melanoma were minimal methylated at this locus [120-122]. Whereas, the islands from cancer cells derived from stage three or more advanced metastatic melanoma samples were hyper-methylated [123,124]. The tumor suppressor lncRNAs will be down-regulated or silenced by DNA methylation. And hence consequent up-regulation of oncogens would be involved in carcinogenesis [125]. Hyper-methylated lncRNAs were re-expressed by demethylation treatment with DNA methylation transferase (DNMT) inhibitor, 5-azadC, within 24-96 h [126]. The expression of hyper-methylated lncRNAs would be further enhanced by treatment in combination with histone deacetylase (HDAC) inhibitor such as 4-phenylbutyric acid or trichostatin [127]. The fact indicated a collaborative role between DNA methylation and histone modifications during the silencing effect of tumor suppressive lncRNAs [128]. A few DNA methyltransferase proteins including Dnmt3a and Dnmt3b [129], as well as methyl-DNA-binding domain proteins (MBDs), are able to form DNA-protein complexes [130].

As for human melanomas, abnormal methylation of the tumor suppressor RASSF1 is a hallmark of many cancers including uveal and metastatic melanoma [131,132]. DNA methylation was considered as predictors of recurrence in non muscle invasive bladder cancer: an MS-MLPA approach [133,134].

Considering the complex origin of melanoma and the existence of heterogeneous subtypes, it is considered that the presence of a single biomarker would not be sufficient to make an informed diagnostic decision [135]. The high affinity RNA-binding activity of MBD proteins was recently characterized and it seemed to be different from the methyl CpG DNA binding domain protein. It was hypothesized that DNMTs and MBD proteins may allow RNA molecules to participate in DNA methylation-mediated chromatin regulation [136].

Chromatin modifications appear to be correlated with CpG island methylation, in which methylation is repeatedly exhibited in tracts of DNA sequence at the fifth carbon atom of cytosines. Cytosine methylation is the only known endogenous modification of DNA in mammals and it occurs through DNA methyltransferase-mediated methylation [137].

There is one of the best understood mechanisms behind epigenetics. It involved methylation of cytosine residues at specific positions in the DNA molecule [138,139]. It has well characterized the enzymes that have carried out the methylation reaction [140]. The mechanism is that the configuration of methylated positions is propagated through DNA replication [141]. The typical consequence of methylation in a genomic region is the repression of nearby genes [142].

### Epigenetic role for lncRNAs in gene regulation

A novel mechanism of epigenetic repression of the RASSF1A tumor suppressor gene has involved antisense unspliced lncRNA. In this mechanism, the expression of the RASSF1 isoform has been selectively repressed by ANRASSF1, overlapping the antisense transcript in a location-specific manner [143].

During the latent infection of human cytomegalovirus (HCMV) in CD14 (+) and CD34 (+) cells, RNA4.9 interacts with components of the polycomb repression complex (PRC) as well as the MIE promoter region where the enrichment of the repressive H3K27me3 mark. It will also disclose the repression function of lncRNA on transcription [144].

Berghoff EG and his colleague have shown that Evf2 (Dlx6as) lncRNA antisense transcription, Evf2-dependent balanced recruitment of activator and repressor proteins enabled differentially transcriptional control of adjacent genes with shared DNA regulatory elements [145]. Researches from another lab indicated that the intronic long non-coding RNA ANRASSF1 recruited PRC2 to the RASSF1A promoter, reducing the expression of RASSF1A and increasing cell proliferation [146]. LncRNA loc285194 is a p53 transcription target; tumor cell growth is inhibited by ectopic expression of loc285194 both *in vitro* and *in vivo*[147].

The lncRNA-LET has been reduced by hypoxia-induced histone deacetylase 3 by reducing the associated histone acetylation-mediated modulation of the lncRNA-LET promoter region. And the down-regulation of lncRNA-LET was found to be a key step in the stabilization of nuclear factor 90 protein. It leads to hypoxia-induced cancer cell invasion [148]. lncRNA-HEIH plays a key role in cell cycle arrest at stage G(0)/G(1). In addition, it was associated with enhancer of zeste homolog 2 (EZH2) and also required for the repression of EZH2 target genes [149].

TNFα expression is regulated by the long non-coding RNA THRIL (TNFα and hnRNPL related immunoregulatory LincRNA: large intergenic non-coding RNAs) through its interaction with hnRNPL (heterogeneous nuclear ribonucleoprotein L) [150]. Both activation and repression of immune response genes would be mediated by lincRNA-Cox2 [151].

### Epigenetic role for lncRNAs in gene activation

The dynamics of miRNA regulatory network mediated by RNA editing is implicated in stroke. LncRNAs-151 is found to be unregulated after middle cerebral artery occlusion. The immature form of lncRNAs-151 is subject to RNA editing that influences the primary lncRNAs processing into mature lncRNAs within the CNS [152]. Intriguingly, lncRNAs-151 is thought to target in various cell cycle regulators as well as protein tyrosine kinase 2 (focal adhesion kinase), which is a non-receptor tyrosine kinase involved in integrin and growth factor signaling pathways. The pathways aredifferentially regulated after middle cerebral artery occlusion and implicated in modulating neurite outgrowth, neuronal plasticity, and restoration of neural network integrity within the ischemic penumbra [153-155]. Furthermore, lncRNADQ786243 makes effects on regulating the expression of CREB and Foxp3, consequently with the regulation of T regulator cells in Crohn’s disease [156].

### Enhancer-like activity of lncRNAs

Enhancer-associated (elncRNA) and promoter-associated (plncRNA) elements play different roles in the chromatin status at intergenic lncRNAs transcription [157]. Expression of elncRNAs, but not plncRNAs, is associated with enhanced expression of neighboring protein-coding genes during erythropoiesis [157].

LncRNAs are dynamically expressed during erythropoiesis with epigenetic regulation. And they are targeted by key erythroid transcription factors such as GATA1, TAL1 and KLF1. After exploring 12 candidate lncRNAs, they were nuclear-localized, exhibiting complex developmental expression patterns. Depleting them severely impaired erythrocyte maturation, inhibiting cell size reduction and subsequent enucleation. lncRNA-EC7 is transcribed from an enhancer and is specifically needed for activation of the neighboring gene encoding BAND 3 [158].

Recently, researchers have identified a translational regulatory lncRNA (trlncRNA) through genome-wide computational analysis. Furthermore, they found trlncRNA was upregulated in paired clinical breast cancer primary and lymph-node metastasis samples. Tumor invasion and metastasis will be stimulated by its expression in vitro and in vivo, respectively. In addition to this, trlncRNA is involved in the down-regulation of the epithelial marker E-cadherin by suppressing the translation of its mRNA [159].

### The epigenetic influence on chromatin from lncRNAs

Cellular reprogramming is known to accompany cell type-specific epigenetic alterations of the genome. It is the conversion of one specific cell type to another. Chromatin structure and dynamics can be influenced by epigenetic factors such as covalent histone modifications, histone variants, DNA methylation, ncRNAs and etc. Chromatin remodeling complex may play an important role in cell fate decision [160]. It has found that 28 lncRNAs are associated in cell invasiveness. It also represented the first key step for successful metastasis. Moreover, another ncRNA (HOTAIR long ncRNA) is able to promote cancer metastasis by inducing epigenetic variations in the chromatin state of cancer cells [161]. Many tumor suppressor genes were found to carry antisense transcripts [162]. For example, p15, a cyclin-dependent kinase inhibitor implicated in leukemia, possesses an antisense transcript and silencing its transcription in *cis* and in *trans* by inducing heterochromatin formation without changing DNA methylation in a Dicer-independent manner [163-166]. It is possible that these antisense transcripts directly bind and recruit chromatin-modifying complexes to their associated sense transcripts [167]. The role of non-coding RNAs in chromatin formation has also been observed in plants [168,169]. One study found that targeted 3 prime processing of a non-coding antisense transcript to the FLC gene (a major floral repressor gene), resulting in the recruitment of FLD. It is a homolog of the human histone demethylase LSD1, which targets H3K4me2 for demethyaltion [170]. Antisense mediated chromatin modifications appear to mostly operate in *cis* in contrast to lincRNAs which can operate both in *cis* and in *trans*[171,172].

The human body is composed of hundreds of distinct cell types. There is a specific position for each cell within the body and each cell performs a specific function. Since all the cells within a multi-cellular organism contain the same genome, the information that inducing cells to establish their identity is likely to be coded in their epigenome [173]. The epigenome is comprised of modifications of DNA (i.e., DNA methylation) and modifications of histone proteins at specific amino acid residues (e.g., acetylation, methylation, phosphorylation, etc.) [174]. Key regulators of the epigenome are chromatin-modifying complexes that can add or remove covalent modifications to chromatin [175,176]. The transcription factors can recognize and bind to specific DNA sequences. In contrast to transcription factors, the majority of chromatin-modifying complexes do not able to binding DNA [177]. A major gap in our understanding of epigenetic regulation for chromatin-modifying complexes is how these complexes are targeted to specific regions of the genome. Recent studies have recently shown that the Jumanji protein Jarid2 could recruit the polycomb repressive complex (PRC)2 to its target sites in mouse embryonic stem cells. However, it showed a low expression in differentiated cells. Therefore, it is not clear how PRC2 is targeted to its genomic sites in other cell types [178]. Also, there is a plethora of chromatin-modifying complexes without DNA binding protein partners to guide them to their action sites [179,180].

**Figure 5:**
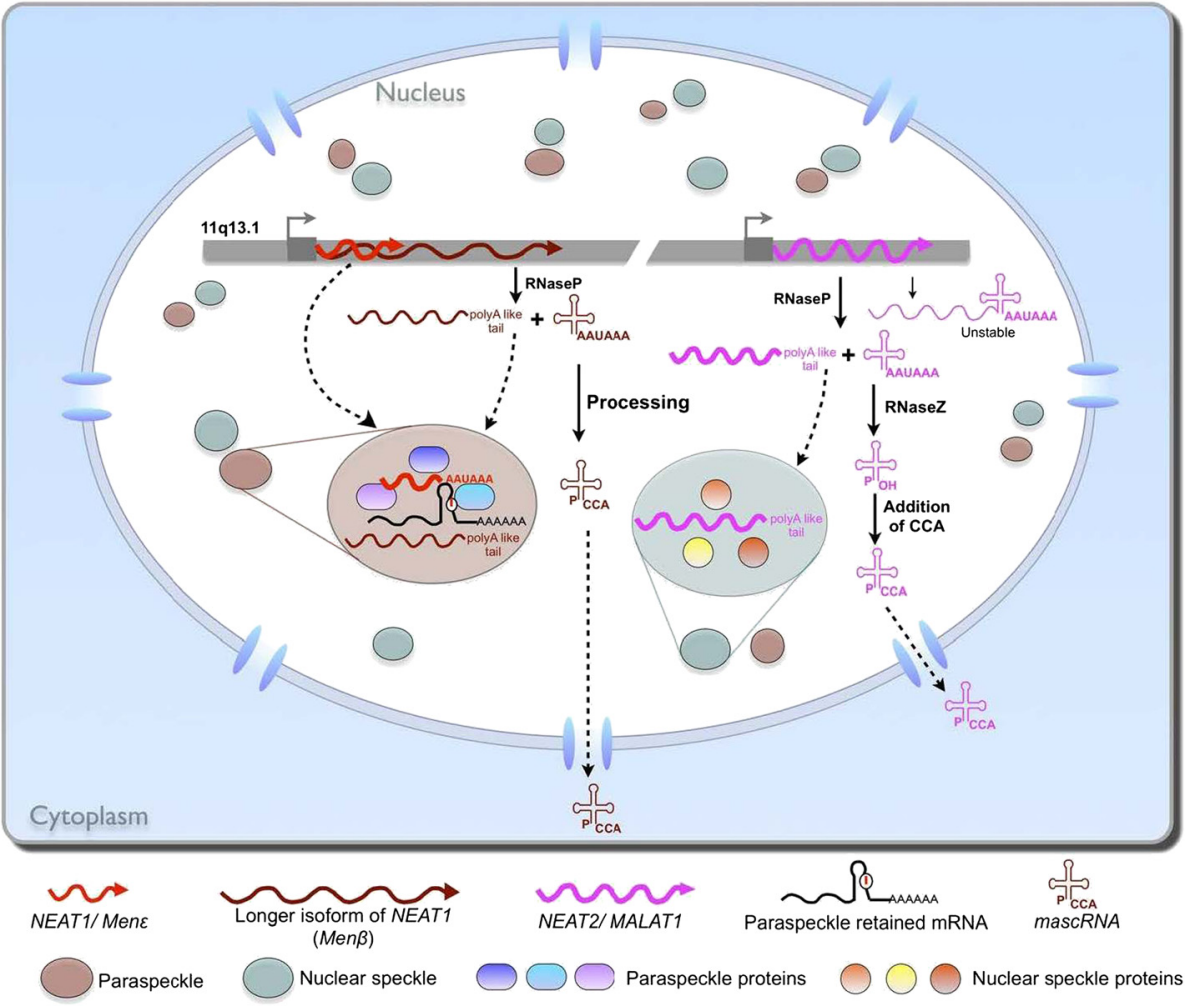
**Long non-coding RNAs in nuclear sub-compartments.** Human *NEAT1* (*Menϵ*in mouse) localizes to para-speckles and it is required for para-speckle structural integrity. *NEAT2* (*MALAT1*) localizes to splicing speckles but it is not required for their structural integrity. Nascent *Menβ* and *MALAT1* transcripts can each be processed by the unusual mechanism of RNase P cleavage to generate the 5’ end of *mascRNA* (*MALAT1*- associated small cytoplasmic RNA) and the 3’ end of the mature *Menβ* and *MALAT1* transcripts, which localize to paraspeckles and splicing speckles, respectively.

Similarly, several large lncRNAs transcribed antisense of protein-coding genes and they can also interact with chromatin modifying complexes and affect the landscape of chromatin [181,182]. For example, the antisense transcript to the Igf2r gene is known as Air and it is required for the allele-specific silencing of several genes in the mouse placenta. The gene functions through direct interaction with the repressive histone methyltransferase G9a [91,183]. Similarly, there is a nuclear retained antisense transcript in Kcnq1 gene (Kcnq1ot1). It associates in a tissue-specific manner with the Chromatin complexes G9a and PRC2 and several protein-coding genes within a 1 Mb region in *cis* will be repressed [31,97] (Figure 5).

### LncRNAs with epigenetic regulation

The mRNA of BCL2 will be negatively regulated by the miR-15a/16-1 group [184]. As an anti-apoptotic gene in cancer, the frequent down-regulation of BCL2 suggests that the failure to induce apoptosis may be reason of melanoma development [185].

LncRNAs play critical roles in epigenetic modulation of chromatin structure by regulating key genes in specific cancerous cells [186,187]. Distinct chromatin signatures are associated with lncRNAs encoding genes, and these signatures are demonstrably different in cancer cells, such as in colorectal carcinoma [188]. It found that the expression of elncRNA, instead of plncRNA, was associated with enhanced expression of neighboring protein-coding genes during erythropoiesis [157]. The regulation of lncRNA gene maternally encoded gene 3 by miR-29 and modulating the corresponding chromatin structures in hepatocellular carcinoma cells [189]. DNMT-3A and DNMT-3B are direct targets of miR-29. It makes effects indirectly through the latter’s influences on DNMT gene expression [190,191]. What is more, over-expressed lncRNAs can be potentially served as a required component of castration-resistance in prostatic tumors with Chromatin remodeling proteins such as Bmi1, Ring2 and Ezh2 [192,193].

The significance of epigenetic regulation of lncRNAs in human melanoma cells is increasing with more evidence [115,192]. LncRNAs was widely studied in such cancers as melanoma, colorectal, head and neck cancer [183]. And LncRNAs clusters were differentially expressed in ovarian cancer cells with varying metastatic potentials. 4,956 lncRNAs have been detected in the microarray, 583 and 578 lncRNAs were upregulated and down-regulated, respectively. Seven of the analyzed lncRNAs (MALAT1, H19, UCA1, CCAT1, LOC645249, LOC100128881, and LOC100292680) confirmed the deregulation found by microarray analysis. LncRNAs play a partial or key role in epithelial ovarian cancer metastasis [194].

## Conclusions and future directions

lncRNAs function make effects in many biological and pathological processes such as stem cell pluripotency, neurogenesis, oncogenesis and etc. In this review, it has focused on the functional roles of lncRNAs in epigenetics and summarized related research progress.

Reasoning, primary ncRNA precursor chains have a high frequency of nonsense codons in their short and highly interrupted ‘reading frames’. In addition, they will never be translated into proteins because they are too short. However, they may be associated with proteins that detect nonsense codons within a reading frame.

Thus, distinct forms of chromatin proteins may make effects through protein–protein contacts via the nonsense-mediated decay complex proteins. It might be able to organize in genes encoding ncRNA or lncRNAs. Chromatin will also be associated with a variety of other RNA binding proteins. In principle, ncRNAs could exert regulatory effects on the chromatin through their association with any of these proteins. Thus, much more exploratory work is needed in these fields.It was indicated that several ncRNAs are functional and not just ‘transcriptional noise’ as has been previously speculated. To the early geneticists, a ‘gene’ was a very abstract entity. It was only considered to reflect the way phenotypes were observed when transmitted between generations. Today, however, it is dispensable to re-evaluate the way for classifying ‘gene’ and genomic regions of apparently ‘gene poor’. It may produce important transcripts. All these will need to be tested with various methods for proving its clinical linkage to diseases.

## Abbreviations

Anril: Antisense non-coding RNA in the ink4 locus; DNMT: DNA methylation transferase; ElncRNA: Enhancer-associated Long non-coding RNA; FANTOM: Functional Annotation of Mammalian cDNA; GRIM–19: Genes associated with retinoid/interferon-induced mortality-19; ICRs: Imprinting control regions; Kcnq1: Potassium voltage-gated channel subfamily Q member 1; LIT1: Long QT intronic transcript 1; LncRNA: Long non-coding RNA; MBD: Methyl-DNA-binding domain proteins; MBD1: Methyl-DNA-binding domain proteins; MEG3: Maternally expressed 3; mRNA: Messenger RNA; ncRNA: Non-coding RNA; ORF: Open reading frame; PlncRNA: Promoter-associated Long non-coding RNA; PRC: Polycomb repressive complex; PRINS: Psoriasis susceptibility-related RNA Gene Induced by Stress; sncRNA: Small non-coding RNAs; SncRNA: Small non-coding RNAs.

## Competing interests

The author’s declare that he has no competing interests.
