# Expression of Concern: The functional role of long non-coding RNAs and epigenetics

**DOI:** 10.1186/s12575-016-0042-1

**Published:** 2016-06-09

**Authors:** Shulin Li

**Affiliations:** Department of Pediatrics-Research, Unit 0853, The University of Texas MD Anderson Cancer Center, Houston, USA

## Expression of Concern

After publication of this article [1] it was brought to the Editors’ attention that the same manuscript had been submitted to another journal by a different author while this article was still under consideration. We have been unable to confirm the authorship despite contacting the authors’ institutions and requesting an investigation.

## References

[1] Cao J (2014). The functional role of long non-coding RNAs and epigenetics. Biological Procedures Online.

